# PRPF19 mRNA Encodes a Small Open Reading Frame That Is Important for Viability of Human Cells

**DOI:** 10.1134/S1607672923700722

**Published:** 2024-03-12

**Authors:** N. M. Shepelev, A. O. Kurochkina, O. A. Dontsova, M. P. Rubtsova

**Affiliations:** 1 Shemyakin-Ovchinnikov Institute of Bioorganic Chemistry of the Russian Academy of Sciences, Moscow, Russia; 2 Department of Chemistry, Moscow State University, Moscow, Russia; 3 Belozersky Institute of Physico-Chemical Biology, Moscow, Russia; 4 Skolkovo Institute of Science and Technology, Center for Molecular and Cellular Biology, Moscow, Russia

**Keywords:** ribosome profiling, upstream ORF, uORF, microprotein, micropeptide

## Abstract

High-throughput ribosome profiling demonstrates the translation of thousands of small open reading frames located in the 5′ untranslated regions of messenger RNAs (upstream ORFs). Upstream ORF can both perform a regulatory function by influencing the translation of the downstream main ORF and encode a small functional protein or microprotein. In this work, we showed that the 5′ untranslated region of the PRPF19 mRNA encodes an upstream ORF that is translated in human cells. Inactivation of this upstream ORF reduces the viability of human cells.

## INTRODUCTION

The emergence of ribosome profiling has made it possible to identify the translation of many RNA molecules and regions of messenger RNAs (mRNAs) that were previously considered non-coding [1]. Subsequent studies revealed the important role of many translation products of “non-coding” RNAs [2–4]. An interesting example of “non-coding” regions of mRNAs is small open reading frames (small ORFs) located in their 5′ untranslated regions (5′ UTR). We refer to them as upstream ORFs hereafter, as established in [1]. The features of the upstream ORF functioning are their ability to regulate gene expression, influencing the translation of the downstream main ORF of the gene, as well as to encode small functional proteins, the role of which may not be directly related to the function of the protein encoded by the gene [5, 6].

One of the known genes containing regulatory upstream ORF is *ATF4*, which encodes a master transcription factor required for cell recovery under stress conditions [7]. The *ATF4* gene mRNA has two upstream ORFs, conserved among mammals, which suppress the translation of the main ORF under normal conditions, but promote its translation under various stresses [8].

An interesting example of an upstream ORF is located in the *ASNSD1* gene involved in asparagine biosynthesis. This upstream ORF encodes a small protein ASDURF [9]. ASDURF is one of 12 subunits of the co-chaperone complex PAQosome [9], which is involved in the biogenesis of several protein complexes [10].

Genome-wide screenings of human genes using the CRISPR-Cas9 system identified the *PRPF19* gene, which is critical for the viability of cells of various lineages [11, 12]. The PRPF19 protein is a highly conserved splicing factor that is included in the Prp19/NTC complex [13]. The main function of this complex is attributed to RNA processing and the response to DNA damage [13]. In this work, to elucidate the role of the upstream ORF in the *PRPF19* gene, we tested whether this upstream ORF could be translated in cells using a reporter construct and studied how its inactivation affects cell viability.

## MATERIALS AND METHODS

### Cell Cultures

HEK293T cells (a derivative line from human embryonic kidney) and HAP1 (a nearly haploid derivative line from human chronic myeloid leukemia) were grown in DMEM/F12 and IMDM (Gibco), respectively, supplemented with alanyl-glutamine (GlutaMAX, Gibco), 10% fetal bovine serum (FBS HI, Gibco) in the presence of 100 units/mL penicillin and 100 μg/mL streptomycin (Gibco) at 37°C and in 5% CO_2_. The confluence of cultures was examined using an inverted microscope. The cells were tested for the absence of mycoplasma (MycoReport, Evrogen).

Cells were transfected according to the manufacturer’s protocol using Lipofectamine 3000 Transfection Reagent (Invitrogen). Using the same reagent, lentiviral particles were prepared according to the manufacturer’s application note.

To analyze competition, cells were transduced with lentiviral particles encoding guide RNA/Cas9 and the green fluorescent protein gene (*EGFP*) with approximately 50% efficiency, and the percentage of surviving cells with EGFP fluorescence was assessed on the MACSQuant Analyzer flow cytometer (Miltenyi Biotec) for several days.

### Rapid Amplification of cDNA 5' Ends (5′RACE)

5′RACE was performed according to the manufacturer’s protocol (5′ RACE System for Rapid Amplification of cDNA Ends, version 2.0, Invitrogen). RNA was isolated from HEK293T cells using the PureLink RNA Mini Kit (Invitrogen). To obtain cDNA and perform subsequent amplification, oligonucleotides 5′-GTCTTCCCTCTCTTCTTGC-3′ and 5′-GGTTAGCACAGTGGCTTTGTC-3′ were used, respectively.

### Plasmids and Constructs

The plasmid for testing the translation of the upstream ORF was made based on the pHaloTag-EGFP vector (Addgene no. 86629), in which the HaloTag sequence was replaced with a sequence corresponding to the 5′ end of the main *PRPF19* mRNA isoform with the insertion of the HiBiT sequence before the stop codon of the upstream ORF.

The plasmid for introducing random mutations using CRISPR-Cas9 was made based on the LeGO-iG2 vector (Addgene no. 27341), in which a sequence from the lentiGuide-Puro vector (Addgene no. 52963) from the U6 promoter to the guide RNA scaffold with a transcription terminator was placed before the SFFV promoter. The guide RNA sequence was cloned into the resulting vector by annealing the oligonucleotides and ligating them into the vector after restriction with BsmBI. The following oligonucleotides were used: 5′-CACCGCTACGCTAGCATCGCTCGGC-3′ and 5′-AAACGCCGAGCGATGCTAGCGTAGC-3′, 5′-CACCGAGCGCTACGCTAGCATCGCT-3′ and 5′-AAACAGCGATGCTAGCGTAGCGCTC-3′ for the upstream ORF *PRPF19*;5′-CACCGCGCGACGACTCAACCTAGTC-3′ and 5′ -AAACGACTAGGTTGAGTCGTCGCGC-3′ for negative control; 5′-CACCGATACGTGCAAATTCACCAGA-3′ and 5′-AAACTCTGGTGAATTTGCACGTATC-3′, 5′-CACCGTGGCCAGGTTGCGGTCGCAG-3′ and 5′-AAACCTGCGACCGCAACCTGGCCAC-3′ for positive controls (for the *PCNA* gene).

### Detection of HiBiT-Mediated Luciferase Activity

To detect luciferase activity resulting from the binding of the large subunit of luciferase LgBiT and the HiBiT peptide, Nano-Glo HiBiT Lytic Detection System (Promega) was used in a 96-well format according to the manufacturer’s protocol. The luminescence intensity (RLU) of each sample was determined using the Victor X5 plate reader (Perkin Elmer).

### Statistical Analysis

Statistical analysis was performed using the GraphPad Prism 6.0 software (GraphPad, USA). Statistical significance was determined using a *t*-test for at least three replicates.

## RESULTS AND DISCUSSION

**Fig. 1.**
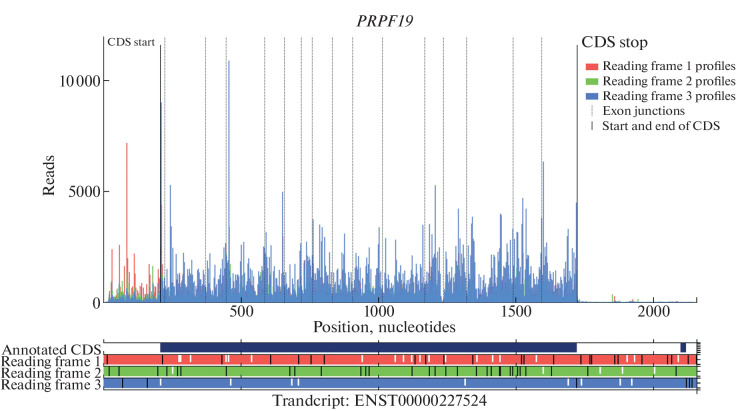
Ribosome profiling of the main mRNA isoform of the human *PRPF19* gene (ENST00000227524) according to the Ensemble database, displayed in the Trips-Viz transcriptome browser. In the diagram below, three possible reading frames and their corresponding profiles on the graph are marked in different colors. In the reading frame diagram, stop codons are marked in black (UGA, UAA, UAG), start codons (AUG) are marked in white.

The main mRNA isoform of the *PRPF19* gene is ENST00000227524 according to the Ensemble database. A simple way to identify ORF in an RNA is to analyze the sequence of interest using the Trips-Viz transcriptome browser [14], which allows to visualize the ribosome profiling data. Using Trips-Viz, it is possible to distribute aggregate ribosome profiling data from different studies into three possible reading frames. To assess which regions of the main isoform of the *PRPF19* gene mRNA are associated with translating ribosomes (Fig. 1), we selected the unambiguous option in Trips-Viz, thus all data displayed should map only to *PRPF19* gene transcripts.

We found that in addition to the annotated main ORF corresponding reading frame 3 marked in blue (Fig. 1), the 5′ UTR of the main mRNA isoform also contains a profile marked in red, which belongs to reading frame 1 (Fig. 1) and contains only one stop codon, which is located at the beginning of the transcript. This observation indicates that the upstream ORF located at the 5′ UTR of the *PRPF19* gene mRNA can be translated. Interestingly, the 5′ UTR lacks the AUG start codon, so the translation of such an upstream ORF must begin with a non-canonical start codon.

**Fig. 2.**
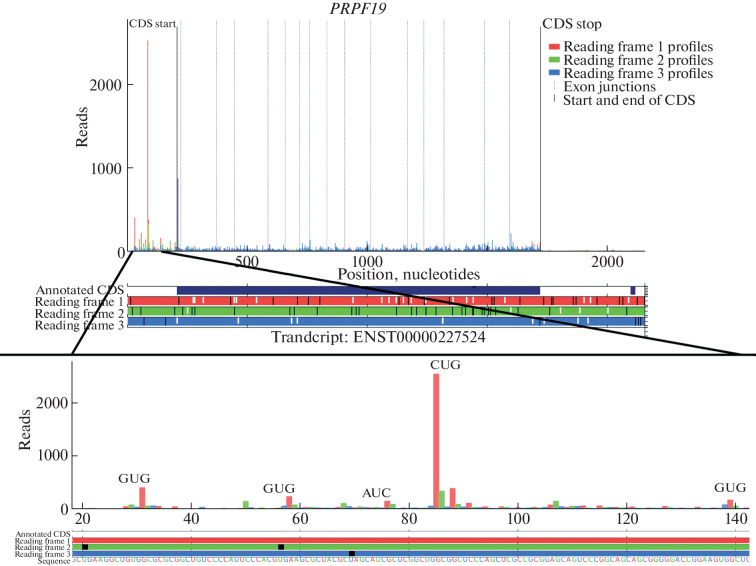
Profiling of initiating ribosomes of the main mRNA isoform of the human *PRPF19* gene (ENST00000227524) according to the Ensemble database, displayed in the Trips-Viz transcriptome browser. In the diagram, three possible reading frames and their corresponding profiles on the graph are marked in different colors. In the diagram below, stop codons (UGA, UAA, UAG) are marked in black, start codons (AUG) are marked in white.

To confirm that the translation of the upstream ORF of the *PRPF19* gene begins with a non-canonical start codon, we selected only Trips-Viz profiling data obtained when ribosomes were stopped at the stage of translation initiation when cells were treated with harringtonine or lactimidomycin (Fig. 2). The resulting aggregate profile of initiating ribosomes from different studies shows that the translation of the upstream ORF can begin with several start codons. Red peaks in the 5′ UTR region of mRNA on the graph correspond to codons CUG, GUG, AUC, which are close to AUG and can be used as start codons during translation initiation [15].

Translation likelihood of the upstream ORF of *PRPF19* gene mRNA is high, as evidenced by the comparable and even higher the number of ribosomes in fraction, especially those initiating on it in comparison with the main ORF. It was necessary to prove that only only one mRNA isoform of the *PRPF19* gene is present in human HAP1 cells. To do this, we performed 5′RACE analysis of mRNA isoforms of the *PRPF19* gene using two oligonucleotides complementary to exons 7 and 8 of *PRPF19* for synthesis of cDNA. Exons 7 and 8 are present in all Ensemble-annotated transcripts related to *PRPF19* gene that contain at least a part of the first exon of the main isoform, where the putative upstream ORF is located. We were able to detect only a sequence that fully matches the main mRNA isoform of the *PRPF19* gene from Ensemble. Thus, ribosome profiling data may belong to only one mRNA of the *PRPF19* gene.

**Fig. 3.**
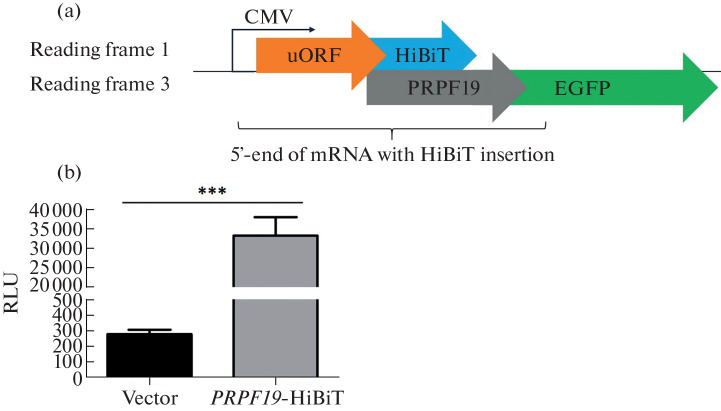
Validation of the upstream ORF translation of the *PRPF19* gene using a reporter construct. (a) Scheme of the reporter construct. Part of the *PRPF19* ORF is joined to the EGFP ORF. uORF: upstream ORF. (b) Measurement of HiBiT-mediated luciferase activity in cell lysates after transfection with reporter constructs. Means and standard deviations for 4 independent transfections are shown. RLU: relative light units; *** *p* value <0.001.

It is necessary to confirm that the upstream ORF may be translated before studying it. To do this, we placed a sequence corresponding to the 5′ end of the *PRPF19* gene mRNA, including the start of the PRPF19 protein ORF, before the green fluorescent protein (EGFP) ORF. In this reporter construct, we added a sequence of an 11-amino acid HiBiT protein tag to the C-terminus of the putative peptide (Fig. 3a). HiBiT makes it possible to detect the presence of a peptide in a sample due to luminescence, which occurs when the inactive LgBiT luciferase subunit is complemented with the HiBiT tag. The original vector without the insertion of the 5′ end sequence of the *PRPF19* gene mRNA was used as a control construct. We transfected HEK293T cells with the resulting constructs and measured HiBiT-mediated luciferase activity (Fig. 3b). The luminescence signal in lysates of cells transfected with the reporter construct was approximately 120-fold higher than the background signal observed in lysates of cells transfected with the control construct (Fig. 3b). Thus, we have shown that the upstream ORF in the 5′ UTR of the *PRPF19* gene mRNA can be translated in human cells in vitro.

**Fig. 4.**
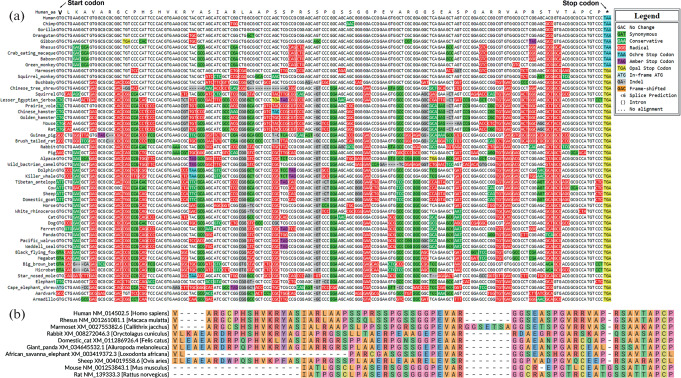
Conservation analysis of the upstream ORF of the *PRPF19* gene among mammals. (a) Alignment of the upstream ORF of the *PRPF19* gene in the CodAlignView genomic browser among selected mammals. The positions of the first start codon of the upstream ORF in humans according to the data in Fig. 2 and stop codon are indicated. (b) Alignment of protein sequences encoded by the upstream ORF in the mRNA of the *PRPF19* gene among the indicated species in MSA4U [17]. The start of the protein is selected by the first possible start codon in the corresponding reading frame.

Thus, we confirmed the translation of the upstream ORF in the human *PRPF19* gene mRNA. We decided to test whether this ORF encodes a small protein. The functionality of a translation product can be indirectly assessed by determining the conservation of the ORF encoding it. To do this, we selected the first putative start codon as the start of the human ORF and aligned the sequences of the upstream ORF in mammals in the CodAlignView genomic browser [16] (Fig. 4a).

We found that the upstream ORF in the *PRPF19* gene mRNA is highly conserved among many mammals, yet a number of non-synonymous substitutions, as well as insertions and deletions, are present that should result in amino acid substitutions in the protein product (Fig. 4a). Moreover, many potential start codons are non-conserved (Fig. 4a), indicating possible evolutionary plasticity in the initiation of translation of this upstream ORF. Therefore, we aligned the sequences of the protein it encodes among several mammals (Fig. 4b). We found that the protein is moderately conserved among mammals generally, but it is more conserved in the C-terminal part. Thus, this small ORF and the protein it encodes may be important for cell function.

**Fig. 5.**
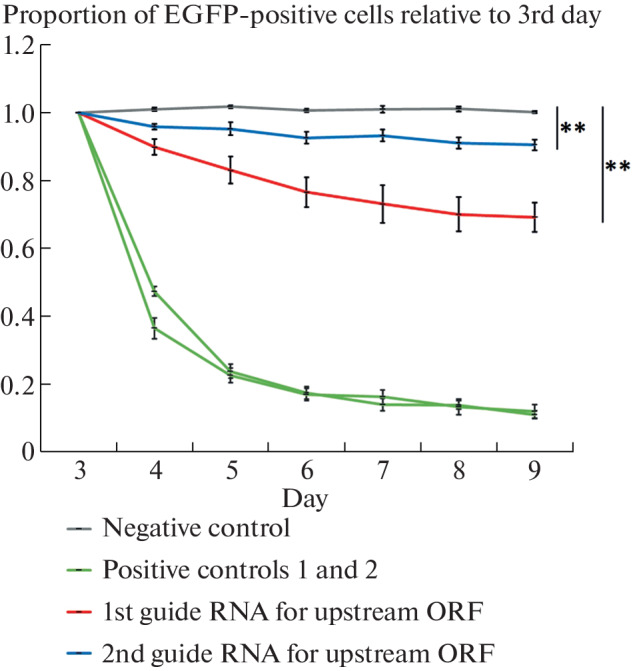
Competition assay of wild-type HAP1 cells and cells expressing guide RNAs over several days. The relative proportion of cells expressing guide RNA and EGFP by day three after lentiviral transduction is shown. Means and standard deviations of three independent cell infections are shown. ** *p* value <0.01.

We next assessed whether mutations in the sequence of this upstream ORF affect cell viability. To do this, we selected the two most specific guide RNAs targeting the middle of the upstream ORF, so that the distance from the start of transcription or from the beginning of the main ORF of the *PRPF19* gene to the nearest cutting site was 71 and 128 nucleotides, respectively, and used them to introduce random mutations using the CRISPR-Cas9 system. Thus, the resulting mutations should not affect the transcription of the *PRPF19* gene or the translation of the main ORF, but will inactivate the upstream ORF by frameshifting. We conducted an experiment to analyze the competition between wild-type cells and cells with a set of random mutations generated in the upstream ORF upon expression of guide RNA (Fig. 5). Guide RNAs targeting the *PCNA* gene, which is necessary for DNA replication, were used as a positive control, and guide RNA, which does not have a complementary region in the genome, was used as a negative control.

We found that expression of guide RNAs that inactivate the upstream ORF in *PRPF19*, but not control guide RNA, reduced cell viability (Fig. 5).

The data obtained in this work indicate that the upstream ORF in the mRNA of the *PRPF19* gene can be translated in human cells in vitro. Moreover, the small protein it encodes is conserved among mammals. Mutations in the upstream ORF lead to the decreased viability of human cells. Thus, either the loss of a small protein or a disruption of the regulatory activity of the upstream ORF leads to a decrease in the viability of human cells.

## CONCLUSIONS

The 5′ untranslated region of the *PRPF19* gene mRNA contains an upstream ORF that is translated in human cells in vitro, as demonstrated using a reporter construct. Inactivation of this upstream ORF reduces the viability of human cells. The conservation of the protein encoded by the upstream ORF in the *PRPF19* gene mRNA among mammals indicates that it may be essential for cell viability. At the same time, it cannot be excluded that this upstream ORF regulates the translation of the main ORF and the level of the PRPF19 protein in the cell, respectively. The regulatory role of the upstream ORF in the *PRPF19* gene mRNA may also be important for cell viability.
